# How Do e-Nutrition Literacy and Faith Shape Positive Nutrition Attitudes? A Machine Learning Approach in Türkiye

**DOI:** 10.3390/nu18030413

**Published:** 2026-01-27

**Authors:** Hande Ongun Yilmaz, Sedat Arslan, Salim Yilmaz

**Affiliations:** 1 Department of Nutrition and Dietetics, Faculty of Health Sciences, Bandirma Onyedi Eylul University, Balikesir 10200, Türkiye; hyilmaz@bandirma.edu.tr; 2 Department of Nutrition and Dietetics, Faculty of Health Sciences, Bursa Uludag University, Bursa 16059, Türkiye; sedatarslan@uludag.edu.tr; 3 Department of Healthcare Management, Faculty of Health Sciences, Acibadem Mehmet Ali Aydinlar University, Istanbul 34752, Türkiye; 4 Department of Healthcare Management, Graduate School of Health Sciences, Acibadem Mehmet Ali Aydinlar University, Istanbul 34752, Türkiye

**Keywords:** e-nutrition literacy, positive nutrition attitudes, religiosity, Türkiye, machine learning, SHAP, Random Forest

## Abstract

**Background/Objectives**: Evidence on religiosity, religious affiliation, and e-nutrition literacy in shaping nutrition attitudes is limited in adult majority-Muslim contexts. The aim of this study is to examine the independent and interactive associations of religiosity, religious affiliation, and e-nutrition literacy with positive nutrition attitudes among adults in Türkiye. **Methods**: This study involved a cross-sectional online survey conducted November–December 2024 via convenience and snowball sampling. After quality checks, 1104 adults remained (mean age = 25.7 years, mean BMI = 23.5 kg/m^2^; 69.3% female, 90.7% Muslim). Religiosity was measured with the Duke University Religion Index, and nutrition literacy and positive nutrition attitudes with validated scales. Demographics and anthropometrics were self-reported. Positive Nutrition Attitudes was the primary outcome, predicted by e-nutrition literacy, analyzed using robust OLS and explored for nonlinearities/interactions with Random Forests and SHAP. A generalized linear model tested three-way interactions of e-nutrition literacy, religious affiliation, and religiosity, adjusting for age and BMI. Performance used train or test splits and five-fold cross-validation. **Results**: e-Nutrition literacy was the strongest predictor (β = 0.155, *p* < 0.001). Cross-validated R^2^ was modest (about 0.04). Random Forests slightly improved fit (test R^2^ about 0.064). SHAP indicated a literacy threshold near 26.1 with predominantly positive contributions above this value. In threshold-stratified models, religiosity showed a positive association (β = 0.332, *p* = 0.010). Non-Muslims had higher unadjusted means, but affiliation effects were not significant after adjustment. **Conclusions**: The results highlight the threshold-dependent role of e-nutrition literacy in positive nutrition attitudes and the independent effect of religiosity. These results suggest that boosting literacy above the critical threshold and incorporating religious values may support healthier nutrition behaviors.

## 1. Introduction

Religious beliefs and practices are among the most salient person-level determinants of health behaviors worldwide [1]. Religiosity and religious affiliation can shape norms related to self-regulation, social support, and health responsibility, while also embedding individuals in communities that transmit dietary expectations, fasting traditions, and food taboos [2]. Evidence linking religiosity to diet quality and weight-related behaviors is mixed across contexts, and appears to vary by the specific religious tradition, the dimension of religiosity assessed, and the outcome under study. Importantly, much of the existing evidence is correlational in nature, limiting causal inferences regarding the direction or mechanisms of these associations. Moreover, prior research has rarely examined how religious influences on nutrition attitudes operate alongside modifiable digital competencies, leaving an important gap in understanding the joint role of values and information-processing skills [3]. In adult majority-Muslim settings, where religious identity is widely shared yet heterogeneously practiced, examining religiosity and affiliation together with modifiable psychological skills may help clarify variability in positive nutrition attitudes—that is, favorable orientations toward adopting healthy eating behaviors [4].

Positive nutrition attitudes refer to favorable orientations toward adopting and maintaining healthy eating behaviors, including intentions to consume nutritious foods, attention to balanced dietary practices, and receptiveness to evidence-based dietary guidance [5]. One such modifiable skill is e-nutrition literacy, a nutrition-specific extension of e-health literacy, defined as the ability to find, appraise, and apply nutrition information encountered in digital environments [6]. As nutrition information ecosystems have migrated to social media and influencer-driven channels, higher e-nutrition literacy may buffer against misinformation, support evidence-seeking, and facilitate favorable attitudes toward dietary guidance [7]. In this respect, e-nutrition literacy represents a potentially critical mechanism through which individuals translate abstract health values into concrete nutrition attitudes. In religious contexts, stronger e-nutrition literacy may further enable individuals to critically evaluate nutrition messages encountered through faith-based networks or religiously framed online content, thereby amplifying value-consistent health behaviors while filtering misleading claims, particularly among those embedded in religious affiliations. Conversely, limited e-nutrition literacy can heighten susceptibility to sensational claims, potentially undermining trust in professional advice and weakening attitudes toward prudent nutrition practices [8]. Prior research has shown that higher e-healthy diet/e-nutrition literacy is associated with healthier eating behaviours and related health outcomes in non-Muslim-majority settings, supporting its role as a central predictor in our model [9]. However, whether this association is strengthened, weakened, or reshaped by religiosity or religious affiliation remains largely unexplored, particularly in adult majority-Muslim populations [10]. Addressing this gap is essential to disentangle whether positive nutrition attitudes arise primarily from digital information skills, from value-based motivations, or from their interaction within specific cultural milieus [11].

Türkiye provides a timely and informative setting for this inquiry. Rapid digitalization has expanded access to nutrition content, while food price volatility and positive healthy nutrition debates keep diet choices salient in daily life [12]. At the same time, the population is predominantly Muslim, yet marked by diverse patterns of religious practice and identity. In such a setting, digital literacy may either reinforce religiously grounded dietary motivations through credible information or exacerbate confusion when misinformation circulates within value-laden contexts. Thus, Türkiye offers a natural context to examine threshold and interaction effects between religiosity, affiliation, and e-nutrition literacy on nutrition attitudes. Mapping how religiosity and affiliation relate to attitudes when e-nutrition literacy is high versus low can reveal leverage points for interventions that respect religious values while upgrading information-navigation skills [13]. More broadly, insights into how digital nutrition literacy interacts with value-based motivations may be transferable to other cultural or religious contexts where beliefs, norms, and social frameworks shape nutrition attitudes. In such settings, integrating information-navigation skills with culturally or faith-informed perspectives may support the development of more contextually responsive nutrition interventions. This integrative perspective—examining religiosity, affiliation, and e-nutrition literacy simultaneously through both classical regression and machine learning approaches—represents a key novelty of the present study.

Accordingly, the primary objective of the current study was to examine both the independent and interactive associations of religiosity, religious affiliation, and e-nutrition literacy with positive nutrition attitudes among adults in Türkiye. Specifically, we tested three hypotheses. First, we hypothesized that higher e-nutrition literacy would be positively associated with positive nutrition attitudes (H_1_). Second, we hypothesized that religiosity would demonstrate an independent positive association with nutrition attitudes, net of digital literacy skills (H_2_). Third, we hypothesized that e-nutrition literacy would moderate the associations of religiosity and religious affiliation with nutrition attitudes (H_3_). By explicitly considering these interaction mechanisms through both traditional regression and machine learning approaches (Random Forest with SHAP interpretation), the study aims to clarify whether interventions should prioritize enhancing e-nutrition literacy, engaging religiously grounded motivations, or strategically combining both approaches to foster durable, positive nutrition attitudes in an adult majority-Muslim context.

## 2. Materials and Methods

### 2.1. Study Design and Setting

We conducted a cross-sectional, web-based survey among adults living in Türkiye between November and December 2024. Recruitment used convenience and snowball sampling via social media platforms and a Google Forms questionnaire. Given the use of convenience and snowball sampling via social media platforms accessible to the researchers, the sample may not fully represent the broader adult population in Türkiye, which should be considered when interpreting the results.

### 2.2. Participants and Eligibility

Eligible participants were ≥18 years, residing in Türkiye, Turkish-speaking, and able to complete an online form. Screening questions excluded individuals reporting an active eating disorder, psychiatric diagnosis, dysphagia or chewing–swallowing problems, a dietitian-guided special diet, food allergy or intolerance, or pregnancy/lactation. Participation was voluntary and uncompensated. Predefined data quality checks were performed and missing data were deleted on a list basis. Before deletion, the dataset contained a total of 1120 observations. Checks revealed 14 individuals whose age was <18, one individual whose weight was incorrectly entered, and one individual whose height was incorrectly entered. A total of 16 rows meeting these criteria were considered missing or incorrectly filled out and were removed. In addition, basic data quality checks (including screening for duplicate entries, response time anomalies, inconsistent answers, and incomplete questionnaires) were conducted, and no further cases met exclusion criteria. Consequently, the post-deletion analytic sample comprised 1104 adults.

### 2.3. Ethics

The protocol complied with the Declaration of Helsinki and received approval from the Bandırma Onyedi Eylül University Non-Interventional Research Ethics Committee (2024-233). Before proceeding, participants viewed an information page and provided electronic informed consent. All survey responses were collected anonymously, stored on password-protected systems accessible only to the research team, and analyzed in de-identified form.

### 2.4. Measures

Sociodemographic and Anthropometric Data: Age, sex, marital status, education, employment, height, and weight were self-reported; BMI was calculated as kg/m^2^.

Religious Affiliation: Participants self-identified as Muslim or non-Muslim (deist, atheist, agnostic, Christian, or other) via a single-choice item.

Religiosity: Religiosity was assessed using the 5-item Duke University Religion Index (DUREL), which captures organizational religiosity (e.g., ‘How often do you attend church or other religious meetings?’), non-organizational religiosity (e.g., ‘How often do you spend time in private religious activities, such as prayer, meditation, or religious study?’), and intrinsic religiosity (e.g., ‘My religious beliefs are what really lie behind my whole approach to life’). Scores range from 5 to 27, with higher scores indicating greater religiosity [14]. The scale demonstrated good internal consistency in the present sample (Cronbach’s α = 0.812). The Turkish survey used the standard DUREL items and anchors.

Positive Nutrition Attitudes: Positive nutrition attitudes were assessed using the Positive Nutrition (PN) subscale of the Attitude Scale for Healthy Nutrition [15]. The full ASHN comprises 21 items across four subscales: Information on Nutrition, Emotion for Nutrition, Positive Nutrition, and Malnutrition. For the present study, we focused on the 5-item Positive Nutrition subscale, which assesses favorable attitudes toward adopting healthy nutritional behaviors (e.g., ‘I try to consume healthy foods,’ ‘I pay attention to having a balanced diet’). Items are rated on a 5-point Likert scale (1 = strongly disagree to 5 = strongly agree), with total scores ranging from 5 to 25. Higher scores indicate more positive attitudes toward healthy nutrition practices. The subscale demonstrated acceptable internal consistency in the present sample (Cronbach’s α = 0.852).

e-Nutrition Literacy: Participants completed the 11-item e-Healthy Diet Literacy Scale, a validated Likert-type instrument capturing the ability to find, evaluate, and apply online nutrition information (e.g., ‘I know how to find helpful information about healthy diet on the Internet,’ ‘I can tell high quality from low quality information about healthy diet on the Internet,’ ‘I have the skills I need to use information from the Internet to improve my daily diet’). Scores range from 11 to 55 (observed range 15–51); higher values indicate greater literacy [9,16]. The scale demonstrated acceptable internal consistency in the present sample (Cronbach’s α = 0.746). A data-driven threshold at 26.1 was later used to classify lower versus higher e-nutrition literacy in interaction models (see Statistical Analysis).

### 2.5. Statistical Analysis

#### 2.5.1. Preliminary Analyses

Prior to hypothesis testing, the psychometric properties of all multi-item scales were evaluated. Internal consistency was assessed using Cronbach’s alpha, and confirmatory factor analysis (CFA) was conducted using maximum likelihood estimation to examine factorial validity. All scales demonstrated acceptable to good internal consistency (α range: 0.746–0.852) and acceptable to excellent model fit (CFI range: 0.935–0.989; RMSEA range: 0.057–0.079); detailed results are provided in the Supplementary Material.

Categorical variables were one-hot encoded. The dependent variable was Positive Nutrition Attitude, while independent variables included religiosity, e-nutrition literacy, age, BMI, and the encoded demographic indicators. The dataset was split into training (80%) and test (20%) sets using stratified sampling to preserve the distribution of religious affiliation.

#### 2.5.2. Linear Regression Models

Model assumptions were assessed as follows: linearity with the RESET and Rainbow tests; homoscedasticity with the Breusch–Pagan test; residual normality with the Anderson–Darling test; autocorrelation with the Durbin–Watson statistic; multicollinearity with variance inflation factors (VIFs); and influence with Cook’s distance and leverage. A baseline OLS model was estimated (R^2^, adjusted R^2^, RMSE, MAE, and coefficient statistics reported). To address heteroskedasticity and non-normal residuals, HC1 heteroskedasticity-consistent standard errors were used and compared against classical OLS estimates. Out-of-sample validity was examined with 5-fold cross-validation (R^2^).

#### 2.5.3. Machine Learning Models and Interpretability

To capture potential non-linearities and interactions, we trained Random Forest models. Hyperparameters were tuned using grid search with cross-validation (GridSearchCV, scikit-learn implementation; 108 combinations), followed by random search with cross-validation (RandomizedSearchCV; 150 random combinations, 5-fold cross-validation). The tuned hyperparameters included the number of trees (*n*_estimators: 500–1500), maximum tree depth (max_depth: 3 to unrestricted), minimum samples for splitting (min_samples_split: 2–20), minimum samples per leaf (min_samples_leaf: 1–8), maximum features per split (max_features: sqrt, log2, or 0.3–0.7), and bootstrap sample proportion (max_samples: 0.6–1.0). The optimal configuration used 1500 trees with max_depth = 3, min_samples_leaf = 8, and max_features = 0.5, reflecting a preference for highly regularized, shallow trees to prevent overfitting (see Supplementary Table S3 for full details). We also optimized XGBoost with randomized search (100 iterations) and early stopping; however, Random Forest consistently performed better. All models were implemented with random_state = 42 to ensure reproducibility. The final Random Forest achieved test R^2^ = 0.064 with minimal overfitting (train R^2^ = 0.098, out-of-bag score = 0.038).

To enhance interpretability, SHAP (SHapley Additive exPlanations) was used to decompose predictions into feature-level contributions and explore heterogeneity. SHAP values quantify each feature’s contribution to a prediction: positive SHAP values indicate that the feature increases the predicted outcome (i.e., more favorable nutrition attitudes), whereas negative SHAP values indicate a decrease. Gaussian Mixture Modeling (GMM) was applied to SHAP values to assess bimodality. The 2-component GMM demonstrated superior fit compared to the 1-component model (AIC = 333.92 vs. 577.57), confirming a bimodal distribution.

#### 2.5.4. Threshold and Interaction Analyses

Threshold analysis identified critical cut-points at e-nutrition literacy ≥26.1 (where 75% of observations showed positive SHAP values) and ≥30 (nearly 100% positive). The 26.1 threshold was subsequently used to stratify participants into lower and higher e-nutrition literacy groups for interaction analyses. These thresholds are data-driven and exploratory, requiring external validation.

A Generalized Linear Model (GLM) with Gaussian family and identity link function was fitted with three-way interactions (e-nutrition literacy × religious affiliation × religiosity), controlling for age and BMI, to examine differential effects on nutrition attitudes:

$$\begin{array}{cc} E(Y_{i})=\beta_{0}+\beta_{1} & \left(eNutLitHigh)+\beta_{2}(NonMuslim)+\beta_{3}(Religiosity\right)\\ & +\beta_{4}(eNutLitHigh\times NonMuslim)+\beta_{5}(eNutLitHigh\times Religiosity)\\ & +\beta_{6}(NonMuslim\times Religiosity)\\ & +\beta_{7}(eNutLitHigh\times NonMuslim\times Religiosity)+\beta_{8}(Age)+\beta_{9}(BMI) \end{array}$$

where $Y_{i}\sim \;Gaussian,\;link=identity.$

Unstandardized regression coefficients (*β*) are reported as effect sizes, representing the expected change in Positive Nutrition Attitudes for a one-unit increase in each continuous predictor, holding other variables constant. For categorical predictors, *β* indicates the mean difference relative to the reference category.

All analyses were conducted in Python (v3.11.12; Python Software Foundation, Wilmington, DE, USA) using pandas (v2.2.2; pandas development team, USA), NumPy (v1.26.4; NumPy Developers, USA), scikit-learn (v1.5.0; scikit-learn Developers, Paris, France), XGBoost (v2.0.3; XGBoost Developers, USA), SHAP (v0.45.0; SHAP Developers, USA), statsmodels (v0.14.2; statsmodels Developers, USA), SciPy (v1.13.1; SciPy Developers, USA), semopy (v2.4.10; semopy Developers, Russia), matplotlib (v3.9.0; matplotlib Developers, USA), and seaborn (v0.13.2; seaborn Developers, USA) [17].

## 3. Results

### 3.1. Descriptive Statistics

**Figure 1:**
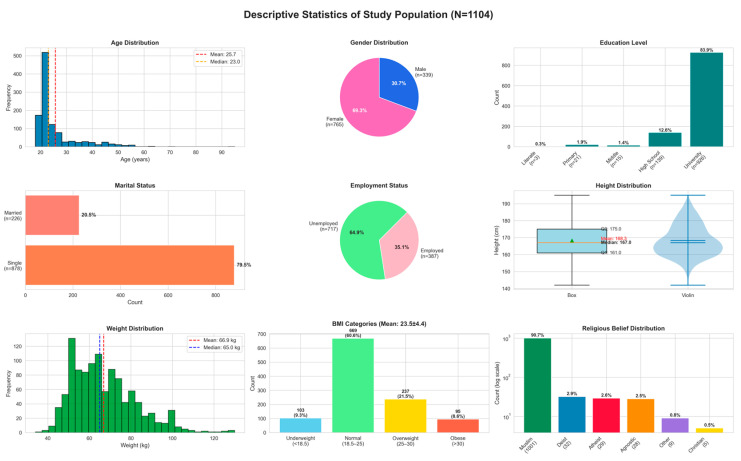
Descriptive characteristics of the study population.

The study included 1104 participants with a mean age of 25.7 ± 8.4 years (median: 23.0, Q1–Q3: 21.0–26.0; range: 18–95). The average height was 168.3 ± 9.2 cm (median: 167.0, Q1–Q3: 161.0–175.0; range: 142–195), mean weight 66.9 ± 15.4 kg (median: 65.0, Q1–Q3: 55.0–75.2; range: 34–130), and mean BMI 23.5 ± 4.4 kg/m^2^ (median: 22.8, Q1–Q3: 20.3–25.8; range: 14.4–45.7). Based on BMI categories, 9.3% were underweight, 60.6% were within the normal range, 21.5% overweight, and 8.6% obese (Figure 1); however, subsequent analyses were conducted using BMI as a continuous variable to capture more detailed variation.

In terms of sociodemographic characteristics, the majority of participants were female (69.3%, n = 765) and single (79.5%, n = 878). Employment status indicated that 64.9% (n = 717) were unemployed. Regarding education, most had a university degree (n = 926, 83.9%), followed by high school graduates (n = 139, 12.6%) and a smaller group with middle school or below (n = 39, 3.5%) (Figure 1).

Descriptive scores for key study variables were as follows: the total religiosity score averaged 16.4 ± 5.1 (range 5–27), positive nutrition attitude averaged 17.2 ± 5.0 (range 5–25), and electronic nutrition literacy averaged 29.9 ± 6.5 (range 15–51). Tests of distributional properties indicated that religiosity (skew = −0.16, kurtosis = −0.52), positive nutrition attitudes (skew = −0.55, kurtosis = −0.26), and e-nutrition literacy (skew = 0.28, kurtosis = −0.24) all demonstrated near-normal distributions. Therefore, no additional transformation of these variables was deemed necessary.

For analysis, these were categorized as University, High School, and Middle or below. With respect to religious belief, 90.7% (n = 1001) identified as Muslim, while 9.3% (n = 103) were classified as Non-Muslim by combining smaller categories (deist, atheist, agnostic, Christian, and other).

### 3.2. Linear Regression Analysis

E-nutrition literacy was the strongest predictor (*β* = 0.155, 95% CI: 0.10–0.21, *p* < 0.001), and high school graduates scored lower than university graduates (*β* = −1.126, 95% CI: −2.17 to −0.08, *p* = 0.034). In contrast, religiosity (*β* = 0.031, *p* = 0.413), religious affiliation (*β* = 0.014, *p* = 0.983), age (*β* = 0.038, *p* = 0.244), and BMI (*β* = 0.002, *p* = 0.955) were not significantly associated with nutrition attitudes. Full coefficient estimates with 95% confidence intervals are provided in the Supplementary Material.

Diagnostic checks identified heteroskedasticity (Breusch–Pagan *p* < 0.05) and non-normal residuals (Anderson–Darling statistic = 6.79, exceeding the 1% critical value), as well as a subset of observations exceeding Cook’s D (6%) and leverage thresholds (7%). Accordingly, HC1 robust standard errors were applied, and substantive conclusions for key predictors remained unchanged. The limited explanatory power of the linear specification, together with assumption violations, motivated the use of Random Forest models with SHAP analyses to explore potential non-linear effects and interactions (see Supplementary Material for full statistical output and diagnostics).

### 3.3. Random Forest and SHAP Analysis

An optimized Random Forest model, tuned through extensive hyperparameter search (150 random combinations with 5-fold cross-validation), achieved only marginal improvement over linear regression (test R^2^ = 0.064, RMSE = 5.01) while maintaining minimal overfitting (train R^2^ = 0.098, test R^2^ = 0.064, OOB score = 0.038). The modest train-test gap (0.034) and the close alignment between OOB and test performance suggest adequate generalization despite the ensemble’s complexity. Feature importance analysis revealed a highly concentrated predictive structure, with e-nutrition literacy accounting for 49.6% of total importance, followed by age (14.6%), religiosity (13.5%), and BMI (13.3%). Permutation importance on the test set confirmed this hierarchy, showing that shuffling e-nutrition literacy led to a substantial performance drop (ΔR^2^ = 0.088), whereas all other predictors had negligible effects (all ΔR^2^ < 0.006). The concentration of predictive power in a single variable motivated the use of SHAP (SHapley Additive exPlanations) analysis to examine individual-level heterogeneity, assess how features contribute to single predictions, and identify potential—albeit limited—non-linear patterns across the data.

**Figure 2:**
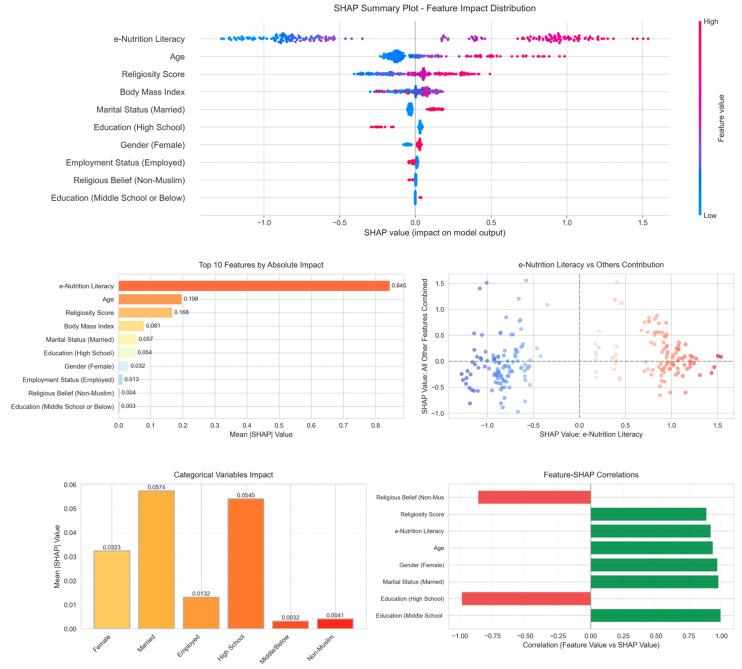
Random Forest Model: Global Feature Importance via SHAP. (**Top panel**)*:* SHAP summary plot displaying the distribution of SHAP values for each feature, with color indicating feature value (red = high, blue = low). (**Middle-left panel**): Mean absolute SHAP values showing e-nutrition literacy as the dominant predictor (0.845). (**Middle-right panel**): Scatter plot comparing e-nutrition literacy SHAP contributions versus all other features combined. (**Bottom-left panel**): Mean absolute SHAP values for categorical variables. (**Bottom-right panel**): Correlations between feature values and their SHAP contributions.

The analysis revealed that e-nutrition literacy’s contribution varied substantially across individuals (SHAP values: −1.28 to 1.54), though with an unexpected bimodal pattern—only 48.9% of observations showed positive SHAP values despite the feature’s overall positive association. The mean absolute SHAP value for e-nutrition literacy (0.845) was 2.63 times larger than the combined contribution of all other predictors (0.322), confirming its dominant but heterogeneous role. Notably, demographic variables demonstrated minimal predictive utility despite their theoretical importance. Gender showed the highest positive rate (71.0% positive SHAP) among categorical variables but with negligible magnitude (mean |SHAP| = 0.032). Educational categories displayed paradoxical patterns, with high school education showing predominantly positive SHAP values (89.6%) despite negative linear coefficients, suggesting complex interactions not captured by main effects. The feature-SHAP correlations revealed strong monotonic relationships for structural variables (education categories showing correlations > 0.98) but weaker associations for continuous predictors, indicating that the Random Forest model primarily used categorical variables for partitioning rather than prediction (Figure 2).

### 3.4. Threshold Effects

To investigate the distributional properties and threshold effects of e-nutrition literacy’s impact, we performed Gaussian Mixture Modeling and threshold analysis on SHAP values. The analysis revealed that e-nutrition literacy’s contribution followed a bimodal distribution, confirmed through Gaussian Mixture Modeling (2-component GMM: AIC = 333.92 vs. 1-component: AIC = 577.57), with distinct clusters around negative and positive SHAP values.

**Figure 3:**
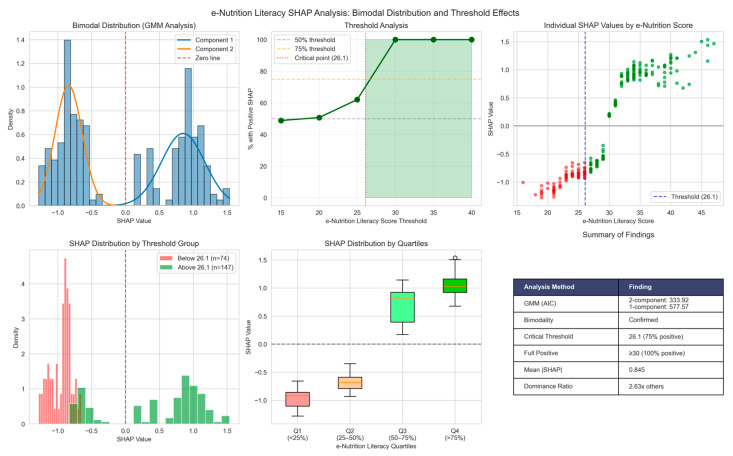
SHAP Distribution Analysis: Bimodality and Threshold Effects in e-Nutrition Literacy. (**Top-left panel**): Gaussian Mixture Modeling confirming bimodal distribution (2-component AIC = 333.92 vs. 1-component AIC = 577.57). (**Top-middle panel**): threshold analysis showing percentage of positive SHAP values at successive e-nutrition literacy scores, with critical cut-point at 26.1 (75% positive). (**Top-right panel**): individual SHAP values plotted against e-nutrition literacy scores, with vertical dashed line indicating the 26.1 threshold. (**Bottom-left panel**): SHAP distribution comparing participants below (n = 74) versus above (n = 147) the threshold. (**Bottom-middle panel**): SHAP values by e-nutrition literacy quartiles. (**Bottom-right panel**): summary table of key results.

As noted before, only 48.9% of observations showed positive SHAP values. Threshold analysis revealed critical cut-points at e-nutrition literacy ≥ 26.1 (75% positive SHAP) and ≥30 (100% positive SHAP). These results suggest that e-nutrition literacy operates through a threshold mechanism rather than linear association, with meaningful benefits emerging only above a score of 26 (Figure 3).

The apparent paradox of high school education showing positive SHAP values (89.6%) was resolved through subgroup analysis: high school graduates actually had lower e-nutrition literacy (28.83 vs. 30.14), older age (30.3 vs. 25.8 years), and negative mean SHAP values (−0.019 vs. 0.006) compared to university graduates, indicating that the tree-based model used education primarily for partitioning rather than direct prediction. This result illustrates an important limitation of tree-based models: variables with high SHAP importance may serve as splitting criteria rather than genuine causal predictors. Education level appears to function as a proxy variable that helps the model partition the feature space to capture complex interactions between age, digital literacy, and nutrition attitudes, rather than directly influencing the outcome. This distinction is crucial for intervention design—improving education levels alone may not enhance nutrition attitudes unless accompanied by improvements in e-nutrition literacy.

**Figure 4:**
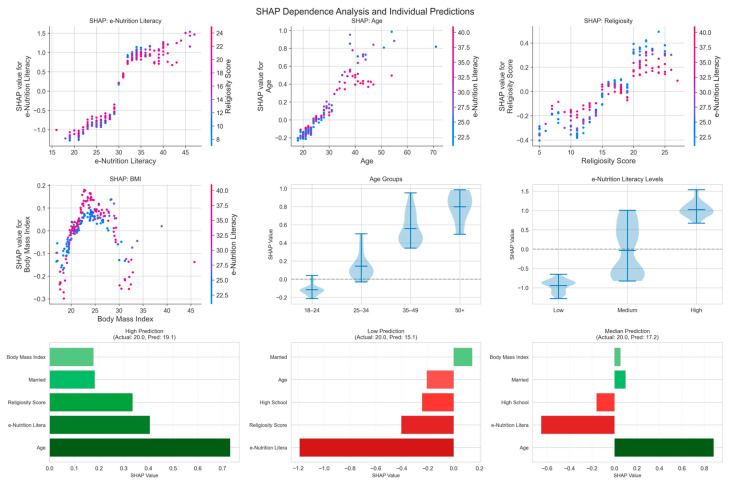
SHAP Dependence and Individual Predictions. (**Top row**): SHAP dependence plots for e-nutrition literacy, age, and religiosity, showing the relationship between feature values (x-axis) and their SHAP contributions (y-axis), with color indicating interaction effects. (**Middle-left panel**): SHAP dependence plot for BMI. (**Middle-center and middle-right panels**): violin plots showing SHAP value distributions across age groups (18–24, 25–34, 35–49, 50+) and e-nutrition literacy levels (low, medium, high), respectively. (**Bottom row**): individual prediction breakdowns for high (Actual: 20.0, Pred: 19.1), low (Actual: 20.0, Pred: 15.1), and median (Actual: 20.0, Pred: 17.2) predicted cases, illustrating feature-level contributions (green = positive, red = negative).

Age demonstrated a clear threshold effect in SHAP values. Younger individuals (under 25 years) had negative SHAP values (mean = −0.127), indicating that being young was associated with lower nutrition attitude scores. In contrast, middle-aged (25–35 years: mean SHAP = 0.098) and particularly older adults showed progressively positive contributions, with those over 50 years having the highest positive impact (mean SHAP = 0.798). This suggests that nutrition awareness and positive attitudes develop with age, possibly due to increased health consciousness, life experience, or health-related concerns that emerge later in life. This non-linear age relationship, which the linear model failed to capture, indicates that nutrition attitudes are influenced by life-stage transitions rather than simply increasing linearly with age. Religiosity and BMI showed mixed effects across individuals. While 54.8% of participants showed positive SHAP values for religiosity and 63.8% for BMI, the remaining participants showed negative values, meaning these variables improved nutrition attitudes for some individuals but worsened them for others. This bidirectional pattern suggests that the relationship between religiosity/BMI and nutrition attitudes depends on unmeasured personal or contextual factors—for instance, religiosity might promote positive nutrition attitudes in some cultural contexts but not others, and BMI’s effect might vary based on individual health awareness or body image concerns (Figure 4).

In the SHAP analysis of the Random Forest model, the observed bimodal distribution and threshold effects indicated that different dynamics may operate below and above the identified cut-off value of 26.1 for e-nutrition literacy. Based on these results, a detailed regression modeling approach was undertaken to systematically examine the differential effects of religion and religiosity on nutrition attitudes within the low and high e-nutrition literacy groups.

**Figure 5:**
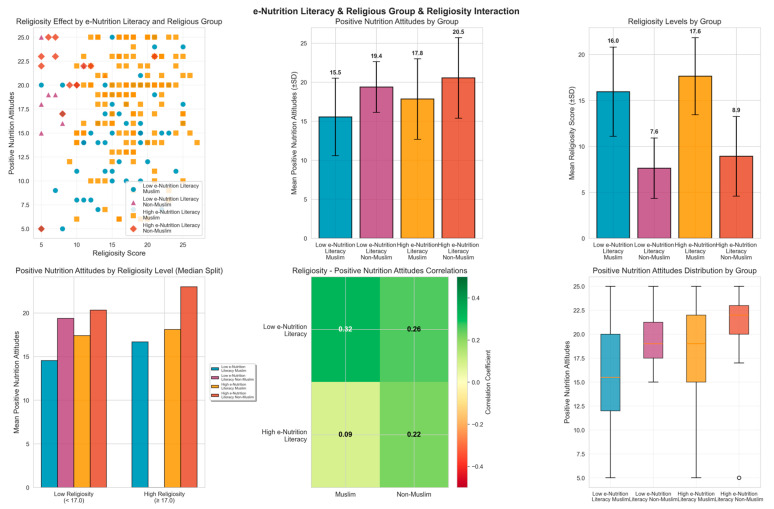
e-Nutrition Literacy, Religious Affiliation, and Religiosity Effects on Positive Nutrition Attitudes. (**Top-left panel**): Scatter plot showing the relationship between religiosity scores and positive nutrition attitudes across four subgroups (Muslims with low e-nutrition literacy, non-Muslims with low e-nutrition literacy, Muslims with high e-nutrition literacy, non-Muslims with high e-nutrition literacy). (**Top-middle panel**): Group means (±SD) for positive nutrition attitudes, with lowest scores in Muslims with low e-nutrition literacy (15.5 ± 4.96) and highest in non-Muslims with high e-nutrition literacy (20.5 ± 5.16). (**Top-right panel**): Group means (±SD) for religiosity scores. (**Bottom-left panel**): Positive nutrition attitudes by religiosity level using median split (17.0). (**Bottom-middle panel**): Correlation heatmap between religiosity and positive nutrition attitudes, with strongest correlation in Muslims with low e-nutrition literacy (r = 0.32) and weakest in Muslims with high e-nutrition literacy (r = 0.09). (**Bottom-right panel**): Box plots showing distribution of positive nutrition attitudes within each subgroup.

Panel A (top-left scatter plot) illustrates the relationship between religiosity scores and positive nutrition attitudes across four subgroups (Muslims with low e-nutrition literacy: blue circles; non-Muslims with low e-nutrition literacy: purple triangles; Muslims with high e-nutrition literacy: orange squares; non-Muslims with high e-nutrition literacy: red diamonds), highlighting differences in distribution between groups. Panels B and C (top-middle and top-right) present bar charts with standard deviation bars for group means of positive nutrition attitudes and religiosity, respectively, showing that the Muslim group with low e-nutrition literacy had the lowest mean positive nutrition attitudes (15.5 ± 4.96), whereas the non-Muslim group with high e-nutrition literacy exhibited the highest values (20.5 ± 5.16). Panel D (bottom-left) compares subgroup means of positive nutrition attitudes using a binary classification of religiosity based on the median value (17.0), revealing that groups with higher religiosity generally demonstrated more favorable nutrition attitudes. Panel E (bottom-middle) visualizes correlation coefficients between religiosity and positive nutrition attitudes in a color-coded heatmap, indicating the strongest correlation in the Muslim group with low e-nutrition literacy (r = 0.32) and the weakest in the Muslim group with high e-nutrition literacy (r = 0.09). Finally, Panel F (bottom-right) displays the distribution of positive nutrition attitudes within each subgroup using box plots, illustrating median values, interquartile ranges, and outliers, thereby highlighting differences in within-group variability (Figure 5).

### 3.5. GLM Interaction Analysis

Based on the identified threshold, participants were stratified into low and high e-nutrition literacy groups using the cut-off score of 26.1. A Generalized Linear Model examined the interactions of religious affiliation (Muslim vs. Non-Muslim) and religiosity with positive nutrition attitudes (Figure 5, Table 1).

Using the cut-off score of 26.1 for e-nutrition literacy, identified as the threshold for achieving positive nutrition, participants were classified into low and high e-nutrition literacy groups. Within this framework, the interactions of religious affiliation (Muslim vs. non-Muslim) and religiosity levels were examined with respect to the dependent variable of positive nutrition attitudes. The GLM results indicated that e-nutrition literacy had a strong and borderline significant main effect on positive nutrition attitudes (β = 5.588, *p* = 0.050), suggesting that individuals with high e-nutrition literacy scored on average 5.59 points higher in positive nutrition attitudes compared to those with lower levels. Similarly, religiosity showed a statistically significant and consistent effect (β = 0.332, *p* = 0.010), indicating that each one-point increase in religiosity score was associated with a 0.33-point improvement in positive nutrition attitudes. However, the interactions between e-nutrition literacy and religious affiliation (*p* = 0.494), e-nutrition literacy and religiosity (*p* = 0.201), religious affiliation and religiosity (*p* = 0.968), as well as the three-way interaction (*p* = 0.774), did not reach statistical significance (Table 1).

Group-based comparisons showed that while Muslims with low e-nutrition literacy had a mean positive nutrition attitude score of 15.55 ± 4.96, non-Muslims at the same literacy level scored higher at 19.38 ± 3.25. In the high e-nutrition literacy group, Muslims had a mean of 17.84 ± 5.15, whereas non-Muslims scored 20.54 ± 5.16. These results indicate that the effects of e-nutrition literacy and religiosity on positive nutrition attitudes are independent rather than synergistic, with both factors contributing significantly on their own. The model explained 11.6% of the total variance (Pseudo R^2^ = 0.116), and given that control variables such as age and BMI did not show significant effects (*p* = 0.957 and *p* = 0.176, respectively), it can be concluded that e-nutrition literacy and religious values play independent yet nearly equally important roles in shaping nutrition attitudes (Table 1).

### 3.6. Summary of Key Results

In plain terms, our analyses revealed three main results. First, e-nutrition literacy was by far the strongest predictor of positive nutrition attitudes—individuals who scored above the threshold of 26 on the e-nutrition literacy scale (approximately the 33rd percentile; roughly one-third of the sample fell below this threshold) scored, on average, 5.6 points higher in positive nutrition attitudes compared to those below. Given that the scale ranges from 5 to 25, this difference represents approximately 28% of the total scale range, indicating a substantial practical effect. Second, religiosity independently contributed to more favorable nutrition attitudes: each one-point increase in religiosity was associated with a 0.33-point increase in positive nutrition attitudes, regardless of e-nutrition literacy level or religious affiliation. Third, contrary to our expectations, we found no evidence that religiosity and e-nutrition literacy work together synergistically; rather, their effects appear to be independent and additive. These results suggest that interventions aimed at improving nutrition attitudes could target e-nutrition literacy skills (particularly raising individuals above the ~26 threshold) and engage religious values separately, without needing to tailor one approach based on the other.

## 4. Discussion

This study shows that e-nutrition literacy is the dominant determinant of positive nutrition attitudes, with clear threshold behavior rather than a purely linear pattern. Across specifications, e-nutrition literacy was the strongest OLS predictor and concentrated nearly half of the Random Forest importance. To enhance interpretability of the Random Forest model, we employed SHAP, a method that decomposes each prediction into feature-level contributions—positive SHAP values indicate that a feature increases the predicted outcome, while negative values indicate a decrease.

SHAP analyses revealed cut-points near 26.1 and 30.0, above which the contribution of e-nutrition literacy was predominantly or entirely positive. Below the functional threshold, individuals can access nutrition information without the appraisal and application skills needed to translate it into favorable attitudes. Once the threshold is crossed, the ability to identify trustworthy sources, reject myths, and plan actions likely accelerates attitudinal gains, consistent with digital health literacy theory linking search, appraisal, and application skills to behaviorally relevant cognitions [18,19,20]. In practical terms, this suggests that merely increasing exposure to nutrition information is insufficient; individuals must reach a minimum competency level before digital content yields meaningful attitudinal benefits. In online ecosystems characterized by variable quality and misinformation, this skill gradient is to be expected [21,22]. Religiosity may operate partly through internalised norms that shape health-related goals and self-regulation; however, the strength and direction of this influence may differ across individuals. For example, individuals with higher intrinsic religiosity (i.e., faith as an internalised commitment) may be more likely to translate value-consistent motivations into stable nutrition attitudes, whereas extrinsic religiosity (i.e., faith motivated primarily by social approval or external demands) may be less consistently related to health-oriented attitudes. In addition, socio-cultural support (e.g., family and community norms, supportive peer networks, and access to trusted health information sources within religious communities) may amplify or buffer the association between religiosity and nutrition attitudes. The heterogeneity observed in SHAP dependence plots underscores this variability, showing that the same level of religiosity can correspond to different attitudinal outcomes depending on individuals’ digital skills and contextual constraints. Consistent with this individual-level variability, the observed SHAP heterogeneity may reflect psychological and environmental moderators such as digital access and skills, exposure to nutrition misinformation, trust in professional guidance, perceived social norms, and broader socio-economic constraints. Future longitudinal and intervention studies are needed to test these moderation hypotheses directly.

Our results are broadly consistent with evidence suggesting that higher digital nutrition literacy is linked to more favorable nutrition-related attitudes and behaviors across settings. For example, population evidence from Taiwan has linked e-healthy diet literacy to health-related behaviors and outcomes [9], supporting the relevance of this construct beyond a single cultural milieu. In Türkiye, studies have supported the reliability and validity of Turkish adaptations of the e-Healthy Diet Literacy Scale and documented meaningful associations with related literacy constructs and participant characteristics [23]. Moreover, Turkish empirical work examining e-nutrition literacy alongside food label reading and positive nutrition attitudes has reported systematic variation across age groups, reinforcing the practical relevance of digital nutrition literacy for adult nutrition attitudes [24]. By leveraging SHAP to visualize non-linear and threshold-like effects, the present study extends this literature by revealing how digital nutrition literacy may function less as a gradual continuum and more as a foundational prerequisite skill that enables other motivational factors to exert influence. Within a majority-Muslim adult sample, our study extends this literature by jointly modeling e-nutrition literacy and religiosity in the same explanatory framework. At the same time, the threshold-like pattern suggested by SHAP should be interpreted cautiously: prior studies typically treat literacy effects as continuous gradients rather than discrete cut-points, so the cut-points observed here are best viewed as exploratory and hypothesis-generating, pending replication and external validation.

Religiosity demonstrated a consistent and independent association with positive attitudes, while neither religious affiliation nor its interactions with e-nutrition literacy or religiosity reached significance in GLMs. This pattern suggests that religiosity may operate through internalized health norms and self-regulatory practices rather than through affiliation-based social boundaries. It aligns with value-congruent health behavior models in which intrinsic religious motivation supports self-care without requiring synergistic effects with information skills [25,26]. Importantly, SHAP results reinforce this interpretation by showing relatively stable, modest contributions of religiosity across the distribution of e-nutrition literacy, indicating additive rather than multiplicative effects at the attitudinal level.

The lack of support for H3—that e-nutrition literacy would moderate the associations of religiosity and affiliation with nutrition attitudes—warrants further consideration. Several factors may explain this null result. First, e-nutrition literacy and religiosity may operate through distinct and parallel pathways: e-nutrition literacy likely influences attitudes through cognitive mechanisms (information appraisal, critical evaluation, and application skills), whereas religiosity may operate through value-based motivations and internalized health norms. These independent pathways would produce additive rather than synergistic effects [27]. Second, the sample’s religious homogeneity (90.7% Muslim) may have limited statistical power to detect interaction effects involving religious affiliation; with only 103 non-Muslim participants, detecting nuanced moderating patterns was likely underpowered Third, ceiling effects may have constrained the detection of interactions: among individuals with high e-nutrition literacy, positive nutrition attitudes were already elevated (mean = 18.1), potentially leaving limited room for religiosity to exert additional moderating influence. Fourth, the threshold-based relationship between e-nutrition literacy and attitudes—rather than a continuous gradient—may have reduced the likelihood of detecting traditional multiplicative interactions. From a theoretical perspective, the independence of these predictors aligns with dual-process models suggesting that skill-based and value-based determinants of health attitudes can function autonomously [28]. Practically, the absence of interaction effects simplifies intervention design: programs can target e-nutrition literacy and engage religious values separately, without needing to tailor literacy interventions based on religiosity levels or vice versa.

Model behavior around education warrants caution. High-school education appeared as a useful splitter in trees despite a negative linear coefficient and only small mean SHAP differences, indicating proxy partitioning of correlated characteristics, such as age, digital skills, and access. This result underscores that feature importance does not equate to causal importance in tree ensembles and that importance can partly reflect partition utility [29]. For policy, the target should be skills rather than credentials: lift e-nutrition literacy directly.

Age exhibited a life-stage pattern: SHAP values were negative under ~25 years and then turned positive, increasing with age. The null linear coefficient in OLS likely reflects this nonlinearity. Younger adults juggle competing priorities and face higher exposure to online misinformation, which can blunt favorable attitudes unless corrected with targeted content and efficacy cues [30]. Accordingly, age-tailored messaging that combines myth debunking with actionable guidance may be particularly important for the under-25 segment.

BMI and most sociodemographic factors had limited predictive utility. BMI showed bidirectional, weak contributions, plausibly reflecting heterogeneity in body image, weight concern, comorbidities, and prior counseling. Small, inconsistent effects are common when distal anthropometrics are linked to proximal attitudes that are more tightly governed by cognitive and behavioral skills such as information appraisal and self-efficacy [31]. Collectively, modest R^2^ and weak demographic effects indicate that a larger share of attitudinal variance likely resides in unmeasured psychosocial and environmental constructs such as norms, media diet, self-efficacy, and food environment.

From a methods standpoint, linearity tests did not reject the multiple-regression specification, yet heteroskedasticity, non-normal residuals, and influential observations were present. Robust HC1 errors mitigated inference issues, and tree models yielded only marginal out-of-sample improvement, indicating limited exploitable nonlinearity in available predictors. These diagnostics support a skills-first intervention logic: rather than searching for complex demographic interactions, move the population above the functional e-nutrition literacy threshold with micro-learning, guided practice, and simple decision aids [19,32,33].

The data support two practical levers. First, set a program target to lift low-literacy individuals above ~26 and, where feasible, closer to 30, since returns appear steeper beyond these points. Second, integrate faith-respectful framing to engage intrinsic motivations associated with religiosity while keeping the curriculum focused on actionable information skills. For youth, add myth-busting modules and low-friction planning prompts.

This study has several limitations that should be taken into account when interpreting the results. This cross-sectional design precludes causal inference, and effect sizes were modest, suggesting that important psychosocial or environmental determinants were unmeasured [19]. In addition, the sample was predominantly female (69%) and highly educated (84% with a university degree), which may have influenced the observed associations and limits the generalisability of the results to more gender-balanced or less-educated adult populations. The sample was young, university-weighted, female-skewed, and predominantly Muslim, reflecting voluntary online recruitment; generalizability is limited beyond similar populations. All constructs were self-reported, raising the possibility of common-method bias and measurement error. The proposed e-nutrition literacy thresholds are data-driven; they require prospective validation before adoption in screening or program targeting. Model diagnostics indicated heteroskedasticity, non-normal residuals, and the presence of influential observations; while robust errors and cross-validation were used, residual model dependence cannot be ruled out [32,33]. Finally, Positive Nutrition Attitudes were operationalized with a brief composite; richer measures of norms, self-efficacy, and media exposure would likely explain additional variance [31]. The modest explained variance across models (R^2^ ≈ 0.04–0.06) suggests that important psychosocial and environmental determinants of nutrition attitudes—such as social norms, media exposure, self-efficacy, and food environment characteristics—remain unmeasured. While this limits predictive utility, the primary aim was to identify relative predictor importance rather than maximize prediction. The proposed e-nutrition literacy thresholds (26.1 and 30.0) are entirely data-driven and exploratory; they require prospective validation in independent samples before any clinical or programmatic application.

In summary, the results indicate that e-nutrition literacy operates with a functional threshold around ~26, above which its contribution to positive nutrition attitudes becomes consistently positive. Religiosity independently supports favorable attitudes, whereas most demographic factors play a comparatively minor role. Together, these results suggest that effective interventions should prioritize strengthening actionable nutrition information skills, complemented by value-aligned messaging to enhance engagement and relevance.

Future research should prospectively validate the data-driven e-nutrition literacy thresholds identified in this study to determine their stability and predictive utility across populations and settings. Experimental or longitudinal designs are also warranted to clarify causal pathways between e-nutrition literacy and positive nutrition attitudes, and to assess whether improvements in literacy translate into sustained attitudinal or behavioral change over time. In addition, incorporating psychosocial and environmental factors—such as self-efficacy, social norms, media exposure, and food environment characteristics—may help explain additional variance and refine intervention targets. From an applied perspective, these results suggest that nutrition policies and programmes could benefit from threshold-informed screening and evaluation approaches, focusing on lifting individuals above functional e-nutrition literacy levels and assessing programme effectiveness not only by exposure but by demonstrated gains in information appraisal and application skills.

Practical implications of the present results suggest several actionable directions for nutrition practice and public health, particularly in digital information environments. First, e-nutrition literacy appears to be a modifiable skill that can be targeted through brief, structured micro-learning modules (e.g., how to verify sources, interpret nutrition claims, and differentiate evidence-based guidance from sensational content) delivered via mobile-friendly formats. Second, in settings where faith-based communities shape health norms, partnering with trusted religious networks and community institutions may help disseminate evidence-informed nutrition messages in value-congruent ways while discouraging misinformation. Third, public health and professional bodies could strengthen “infodemic” mitigation strategies by providing shareable, plain-language content, rapid myth-correction resources, and guidance for influencers/community leaders who communicate nutrition information online. Fourth, given potential disparities in digital access and skills, interventions should prioritize groups that may be at higher risk of low e-nutrition literacy (e.g., older adults, individuals with lower educational attainment, and those with limited digital access), using tailored delivery channels beyond social media when needed. Finally, in clinical and counseling contexts, brief screening of e-nutrition literacy could help identify individuals who may benefit most from targeted education and guided digital navigation support, thereby potentially improving receptivity to dietary guidance and reducing vulnerability to misleading online claims.

## 5. Conclusions

E-nutrition literacy is a dominant and threshold-dependent predictor. The study sample was young, predominantly female, highly educated, and mostly Muslim, suggesting that these results may be most relevant to similar populations and contexts. Cross-sectional analyses indicated that individuals scoring above the e-nutrition literacy threshold (~26) scored, on average, 5.6 points higher in terms of positive nutrition attitudes compared with those below the threshold. Religiosity contributes independently, with a significant positive association (β = 0.33, *p* = 0.01), whereas interactions with affiliation or literacy were not supported. These results indicate that e-nutrition literacy and religiosity may represent separable, potentially actionable correlates of nutrition attitudes; however, whether targeting them independently is effective should be tested in longitudinal and intervention studies. Interventions should lift e-nutrition literacy above ~26 with micro-learning and guided practice, layer faith-respectful messaging to harness intrinsic motivation, and provide age-tailored myth-busting for younger adults. Future studies should validate thresholds prospectively, add granular psychosocial and media-exposure measures, and test scalable strategies that move large segments over the functional literacy threshold while respecting cultural and religious contexts. Targeted efforts to enhance e-nutrition literacy, combined with the culturally sensitive engagement of intrinsic religious motivations, show promise in fostering healthier nutrition attitudes at a population level.

## Figures and Tables

**Table 1:** Generalized Linear Model estimates on Positive Nutrition Attitudes.

Term	β	SE	z	*p*	95% CI
Intercept	12.789	2.778	4.604	<0.001 **	[7.344–18.233]
High e-Nutrition Literacy	5.588	2.848	1.962	0.050 †	[0.006–11.170]
Non-Muslim	6.705	5.184	1.293	0.196	[−3.457–16.866]
High e-Nutrition Literacy × Non-Muslim	−4.391	6.414	−0.685	0.494	[−16.963–8.181]
Religiosity	0.332	0.130	2.562	0.010 *	[0.078–0.587]
High e-Nutrition Literacy × Religiosity	−0.212	0.165	−1.280	0.201	[−0.536–0.112]
Non-Muslim × Religiosity	−0.024	0.589	−0.041	0.968	[−1.178–1.130]
High e-Nutrition Literacy × Non-Muslim × Religion	0.196	0.684	0.287	0.774	[−1.144–1.537]
Age	0.002	0.043	0.053	0.957	[−0.082–0.087]
BMI	−0.115	0.085	−1.353	0.176	[−0.281–0.052]

Log-likelihood = −663.83, Pseudo R^2^ = 0.116; Dependent Variable: Positive Nutrition Attitudes; †: < 0.1; **: < 0.05; *: < 0.001.

## Data Availability

The data presented in this study are available on request from the corresponding author. The data are not publicly available due to ethical and privacy restrictions.

## Supplementary Materials

The following supporting information can be downloaded at: https://www.mdpi.com/article/10.3390/nu18030413/s1, File A. Detailed Statistical Results. Table S1. Out-of-sample performance across models; Table S2: Feature Importance Comparison Across Methods; Table S3. Random Forest Hyperparameter Optimization.

## Abbreviations

The following abbreviations are used in this manuscript:

AIC	Akaike Information Criterion
ASHN	Attitude Scale for Healthy Nutrition
BMI	Body Mass Index
CFA	Confirmatory Factor Analysis
CFI	Comparative Fit Index
DUREL	Duke University Religion Index
GLM	Generalized Linear Model
GMM	Gaussian Mixture Modeling
OLS	Ordinary Least Squares
PN	Positive Nutrition
RMSEA	Root Mean Square Error of Approximation
SHAP	SHapley Additive exPlanations
VIF	Variance Inflation Factor
