# Supplementary material for: How Do e-Nutrition Literacy and Faith Shape Positive Nutrition Attitudes? A Machine Learning Approach in Türkiye
Source: Nutrients. 2026 Jan 27;18(3):413. doi: 10.3390/nu18030413 (PMC12899087; doi:10.3390/nu18030413)
Supplement: Supplementary file 1 [file nutrients-18-00413-s001.zip › nutrients-4099084-SI.pdf]

Supplementary Material

File A. Detailed Statistical Results

Model Specification and Sample Characteristics

A multiple linear regression analysis was conducted using ordinary least squares estimation with positive nutrition attitudes as the continuous dependent variable. The analytical sample comprised 1,104 Turkish adults following listwise deletion of cases with missing data. Train-test split employed stratified sampling based on religious affiliation (80:20 ratio) to ensure representative group distributions across subsets.

Categorical Variable Encoding and Reference Categories

All categorical predictors were dummy-coded using the following reference categories:

- Gender: Male (n = 339, 30.7%) vs. Female (n = 765, 69.3%)
- Marital Status: Single (n = 878, 79.5%) vs. Married (n = 226, 20.5%)
- Employment Status: Unemployed (n = 717, 65.0%) vs. Employed (n = 387, 35.0%)
- Educational Attainment: University (n = 926, 83.9%) vs. High School (n = 139, 12.6%) vs. Middle school or below (n = 39, 3.5%)
- Religious Affiliation: Muslim (n = 1,001, 90.7%) vs. Non-Muslim (n = 103, 9.3%)

University education served as the reference category for educational comparisons, allowing interpretation of coefficients as deviations from the highest educational attainment group. Muslim affiliation served as the reference for religious group comparisons.

Scale Reliability and Confirmatory Factor Analysis

Internal consistency and factorial validity were assessed for all multi-item scales used in the present study. Cronbach's alpha was computed to evaluate internal consistency reliability, and confirmatory factor analysis (CFA) was conducted using maximum likelihood estimation to examine factorial structure.

Duke University Religion Index (DUREL): The 5-item DUREL demonstrated good internal consistency (Cronbach's $\alpha = 0.812$) and good factorial validity ($\chi^2 = 29.27$, df = 3, CFI = 0.989, TLI = 0.963, RMSEA = 0.079). Correlated errors were specified between items with similar content (Durel1–Durel2: organizational religiosity items; Durel4–Durel5: intrinsic religiosity items). All factor loadings were significant and ranged from 0.92 to 1.41.

Positive Nutrition Subscale (ASHN-PN): The 5-item Positive Nutrition subscale demonstrated good internal consistency (Cronbach's $\alpha = 0.852$) and excellent factorial validity ($\chi^2 = 29.45$, df = 5, CFI = 0.989, TLI = 0.978, RMSEA = 0.067). All factor loadings were significant and ranged from 1.00 to 1.11.

e-Nutrition Literacy Scale: The 11-item e-Nutrition Literacy scale demonstrated acceptable internal consistency (Cronbach's $\alpha = 0.746$) and acceptable factorial validity ($\chi^2 = 172.54$, df = 38, CFI = 0.935, TLI = 0.906, RMSEA = 0.057). Correlated errors were specified among semantically similar items (ENLS4–ENLS5–ENLS6: application-related items; ENLS8– ENLS9: evaluation-related items). Factor loadings for core information-seeking items (ENLS1– ENLS3) were strongest (0.75–1.00).

All scales met established thresholds for internal consistency ($\alpha \geq 0.70$) and factorial validity (CFI > 0.90, RMSEA < 0.08), supporting their use in the present analyses.

Complete Statistical Output of Multiple Linear Regression

Model Summary Statistics:

- Multiple $R^2 = 0.0617$
- Adjusted $R^2 = 0.0509$
- $F_{(10,872)} = 5.7297$, $p < 0.001$
- Root Mean Square Error = 5.022
- Mean Absolute Error = 4.099

Coefficient Estimates with 95% Confidence Intervals:

- Intercept: β = 11.065 (SE = 1.397, t = 7.92, p < 0.001, 95% CI: 8.32-13.81)
- E-nutrition Literacy: β = 0.155 (SE = 0.026, t = 5.94, p < 0.001, 95% CI: 0.10-0.21)
- Religiosity: β = 0.031 (SE = 0.038, t = 0.82, p = 0.413, 95% CI: -0.04-0.10)
- Age: β = 0.038 (SE = 0.032, t = 1.17, p = 0.244, 95% CI: -0.03-0.10)
- BMI: β = 0.002 (SE = 0.041, t = 0.06, p = 0.955, 95% CI: -0.08-0.08)
- Female: β = 0.057 (SE = 0.382, t = 0.15, p = 0.881, 95% CI: -0.69-0.81)
- Married: β = 0.567 (SE = 0.629, t = 0.90, p = 0.368, 95% CI: -0.67-1.80)
- Employed: β = -0.244 (SE = 0.371, t = -0.66, p = 0.511, 95% CI: -0.97-0.49)
- High School: β = -1.126 (SE = 0.532, t = -2.12, p = 0.034, 95% CI: -2.17--0.08)
- Middle/Below: β = -0.317 (SE = 1.073, t = -0.30, p = 0.768, 95% CI: -2.42-1.79)
- Non-Muslim: β = 0.014 (SE = 0.663, t = 0.02, p = 0.983, 95% CI: -1.29-1.32)

Multicollinearity Assessment

Variance Inflation Factors indicated absence of problematic multicollinearity among substantive predictors (all VIF < 3.0). The elevated VIF for the intercept (71.97) reflects standard centering effects and does not indicate analytical concerns.

Model Residual Analysis

Model residuals were examined for linear regression assumptions. The Durbin–Watson statistic was 2.13, suggesting no serial correlation. In contrast, the Anderson–Darling test indicated significant departures from normality of residuals (statistic = 6.79, exceeding the 1% critical value = 1.09). These results imply deviations from strict normality, although regression coefficients remain consistent under robust estimation.

Outlier and Influence Diagnostics

Cook's distance values were all < 1.0, but approximately 6% of observations exceeded the 4/n threshold. Leverage diagnostics also indicated that 7.1% of cases surpassed the 2p/n criterion, suggesting the presence of moderately influential observations. Nonetheless, no single case exerted an undue influence on overall model estimates.

Cross-Validation Performance

A 5-fold cross-validation procedure was conducted to evaluate the out-of-sample performance of the baseline regression. The average R^2 across folds was 0.037 (SD = 0.015), consistent with the modest explanatory power observed in the holdout test set.

Robustness to Heteroscedasticity

Breusch–Pagan tests indicated evidence of heteroscedasticity. Therefore, robust standard errors (HC1) were estimated. Results demonstrated that the statistical significance of key predictors, notably E-nutrition Literacy and educational attainment, remained stable after correction, supporting the robustness of the findings.

Robust Standard Errors (HC1)

To address heteroskedasticity identified in diagnostic tests, robust standard errors (HC1, degrees of freedom corrected) were estimated. Results demonstrated that substantive conclusions remained unchanged: E-nutrition Literacy continued to be the strongest predictor of positive nutrition attitudes, and the educational gradient persisted.

- Intercept: $\beta = 11.065$ (SE = 1.407, t = 7.87, $p < 0.001$, 95% CI: 8.30–13.83)
- E-nutrition Literacy: $\beta = 0.155$ (SE = 0.025, t = 6.10, $p < 0.001$, 95% CI: 0.11–0.20)
- Religiosity: $\beta = 0.031$ (SE = 0.039, t = 0.80, p = 0.427, 95% CI: –0.05–0.11)
- Age: $\beta = 0.038$ (SE = 0.030, t = 1.24, p = 0.217, 95% CI: –0.02–0.10)
- BMI: $\beta = 0.002$ (SE = 0.038, t = 0.06, p = 0.951, 95% CI: –0.07–0.08)
- Female: $\beta = 0.057$ (SE = 0.377, t = 0.15, p = 0.880, 95% CI: –0.68–0.80)
- Married: $\beta = 0.567$ (SE = 0.620, t = 0.92, p = 0.362, 95% CI: –0.65–1.78)
- Employed: $\beta = -0.244$ (SE = 0.376, t = –0.65, p = 0.517, 95% CI: –0.98–0.50)
- High School: $\beta = -1.126$ (SE = 0.610, t = –1.85, p = 0.065, 95% CI: –2.32–0.07)
- Middle/Below: $\beta = -0.317$ (SE = 1.025, t = –0.31, p = 0.757, 95% CI: –2.33–1.69)
- Non-Muslim: $\beta = 0.014$ (SE = 0.675, t = 0.02, p = 0.983, 95% CI: –1.31–1.34)

Tree-Based Models (Random Forest and XGBoost)

To explore potential non-linear effects and interactions beyond the linear specification, albeit limited, we estimated tree-based ensemble models using the same train/test split (80/20, stratified by religious affiliation) and the same predictor set as in OLS. Random Forest (RF) was tuned via RandomizedSearchCV in an extended space and evaluated with 5-fold CV. XGBoost was tuned via RandomizedSearchCV (100 iterations) and terminated with early stopping on an internal validation split.

Table S1. Out-of-sample performance across models

Model	Train R^2	Test R^2	RMSE	MAE	CV R^2 (SD)
Linear Regression	0.062	0.060	5.022	4.099	0.037 (0.015)
Random Forest (tuned)	0.098	0.064	5.013	4.097	0.035 (0.013)
XGBoost (early-stopped)	0.094	0.050	5.049	4.146	0.028 (0.013)

All models used identical train/test splits (80/20) stratified by religious affiliation. CV = 5-fold cross-validation.

The performance comparison across models reveals consistent but modest predictive capacity across all specifications. While the Random Forest model achieved marginally superior test performance (R^2=0.064) compared to linear regression (R^2=0.060), the improvement is minimal, suggesting that non-linear relationships and interactions contribute little beyond linear effects. The comparable train-test

gaps across models indicate successful regularization, with Random Forest showing the best balance between model complexity and generalization. The consistently low cross-validation scores (all CV $R^2<0.04$) confirm that the modest predictive power is robust to sample variation rather than an artifact of the specific train-test split.

Table S2. Feature Importance Comparison Across Methods

Feature	Linear β (p-value)	RF Importance (%)	Permutation Importance (ΔR^2)
E-nutrition Literacy	0.155 (<0.001)**	49.6	0.088
Age	0.038 (0.244)	14.6	–0.002
Religiosity	0.031 (0.413)	13.5	0.006
BMI	0.002 (0.955)	13.3	0.001
Education (High School)[a]	–1.126 (0.034)*	2.8	0.003
Marital Status (Married)[b]	0.567 (0.368)	2.4	0.000
Employment (Employed)[c]	–0.244 (0.511)	1.6	0.001
Gender (Female)[d]	0.057 (0.881)	1.5	0.000

* p<0.05; ** p<0.001. Reference categories: [a]University, [b]Single, [c]Unemployed, [d]Male; RF = Random Forest; Permutation importance = mean R^2 decrease on the test set. RF importance represents Gini-based feature importance from the final model. Permutation importance calculated using 50 repetitions on test set.

Feature importance metrics converge on E-nutrition Literacy as the dominant predictor across all methodologies. The Random Forest importance (49.6%) and permutation importance ($\Delta R^2=0.088$) for E-nutrition Literacy far exceed all other predictors combined, reinforcing its central role. Notably, while tree-based models assign moderate importance to age (14.6%), religiosity (13.5%), and BMI (13.3%) through Gini impurity, their permutation importance values are near-zero, suggesting these variables primarily serve as splitting criteria without meaningful predictive contribution. The discrepancy between linear coefficients and tree-based importance for education highlights potential threshold effects captured by tree splits but not linear slopes.

Table S3. Random Forest Hyperparameter Optimization

Parameter	Search Range	Optimal Value
Number of trees	[500–1500]	1500
Maximum tree depth	[3–None]	3
Minimum samples to split	[2–20]	10
Minimum samples per leaf	[1–8]	8
Maximum features per split	{sqrt, log2, 0.3–0.7}	0.5
Maximum bootstrap samples	[0.6–None]	0.8
Bootstrap sampling	True	True

The optimal hyperparameters reveal a preference for highly constrained trees with substantial regularization (min_samples_leaf=8), indicating that deeper, more complex trees led to overfitting. The large ensemble size (1500 trees) combined with feature subsampling and sample subsampling suggests that predictive gains emerged from averaging many weak learners rather than complex individual trees. These conservative parameters align with the limited signal in the data and help explain why the Random Forest model avoided overfitting despite the high dimensionality relative to sample size.
